# In Vitro Evaluation of *Candida albicans* Adhesion on Heat-Cured Resin-Based Dental Composites

**DOI:** 10.3390/ma16175818

**Published:** 2023-08-25

**Authors:** Francesco De Angelis, Simonetta D’Ercole, Mara Di Giulio, Mirco Vadini, Virginia Biferi, Matteo Buonvivere, Lorenzo Vanini, Luigina Cellini, Silvia Di Lodovico, Camillo D’Arcangelo

**Affiliations:** 1 Department of Medical, Oral and Biotechnological Sciences, “G. d’Annunzio” University of Chieti–Pescara, 66100 Chieti, Italy; fda580@gmail.com (F.D.A.); m.vadini@unich.it (M.V.); virg.bif@gmail.com (V.B.); matteo.buonvivere@unich.it (M.B.); camillo.darcangelo@unich.it (C.D.); 2 Department of Pharmacy, “G. d’Annunzio” University of Chieti–Pescara, 66100 Chieti, Italy; mara.digiulio@unich.it (M.D.G.); l.cellini@unich.it (L.C.); silvia.dilodovico@unich.it (S.D.L.); 3 Corso S. Gottardo 25, 6830 Chiasso, Switzerland; vaniniodonto@gmail.com

**Keywords:** microbial adhesion, resin composites, heat-cured, *Candida albicans*, dental biofilm, dental caries

## Abstract

Microbial adhesion on dental restorative materials may jeopardize the restorative treatment long-term outcome. The goal of this in vitro study was to assess *Candida albicans* capability to adhere and form a biofilm on the surface of heat-cured dental composites having different formulations but subjected to identical surface treatments and polymerization protocols. Three commercially available composites were evaluated: GrandioSO (GR), Venus Diamond (VD) and Enamel Plus HRi Biofunction (BF). Cylindrical specimens were prepared for quantitative determination of *C. albicans* S5 planktonic CFU count, sessile cells CFU count and biomass optical density (OD_570 nm_). Qualitative Concanavalin-A assays (for extracellular polymeric substances of a biofilm matrix) and Scanning Electron Microscope (SEM) analyses (for the morphology of sessile colonies) were also performed. Focusing on planktonic CFU count, a slight but not significant reduction was observed with VD as compared to GR. Regarding sessile cells CFU count and biomass OD_570 nm_, a significant increase was observed for VD compared to GR and BF. Concanavalin-A assays and SEM analyses confirmed the quantitative results. Different formulations of commercially available resin composites may differently interact with *C. albicans*. The present results showed a relatively more pronounced antiadhesive effect for BF and GR, with a reduction in sessile cells CFU count and biomass quantification.

## 1. Introduction

Due to their current improvements in mechanical properties and aesthetic performance, dental resin composites (DRCs) have become the most preferred filling material in direct dental restorations. [1,2,3,4,5]. DRCs are typically made out of an organic matrix, inorganic fillers and a silane coupling agent, which connects the two components [6]. The organic matrix consists of several monomers, such as BisGMA (2,2-bis [p-(2’-hidroxy-3’-methacryloxypropoxy) phenyl]- propane), UDMA (urethane dimethacrylate), TEGDMA (trietylenglycol di-methacrylate), DMAEMA (dimethylaminoethyl methacrylate) and various additives (photoinitiators-camphoroquinone, inhibitors, stabilizers). The organic matrix, in the form of a three-dimensional cross-linked network, is created during the polymerization reaction of the monomer mixture of resin composites [7]. The most used technique for starting the polymerization reaction of resin composites is photo-activation [7]. However, monomer conversion is never complete during the polymerization of light-cured resin composites [8] and, according to data from the literature, up to 45% of monomer and double bonds may go unreacted as methacrylate pendant groups [9]. The degree of conversion of double carbon bonds to single carbon bonds achieved during polymerization directly affects the final characteristics of photo-cured composites [10]. When resin composites are used for indirect restorations, before final restoration delivery, they can be further processed in the dental laboratory; here, they are typically subjected to additional heat curing protocols that, according to several studies, allow for enhanced material’s microhardness, flexural strength, fracture toughness, wear resistance, tensile strength and color stability [11,12,13,14]. The conversion of monomers into durable polymer chains is increased as a result of such a heat curing [15,16]. As stated in the literature, in fact, without a post-curing heat treatment process, the polymer matrix is not strong enough, and failure of composites under loading is typically caused by the crushing of the polymer matrix [17]. Many different heat-curing protocols are described in the literature, in which an additional material photopolymerization is performed for a prolonged time (ranging from 10 min to a maximum of 6 h) at an increased temperature (even up to 120 °C) [18,19,20].

Dental materials have been widely investigated in terms of their mechanical performances [21,22,23,24]. At the same time, their biological properties should also be considered of paramount clinical relevance. Oral microorganisms are found in highly structured and ordered microbial communities called biofilms, where microbial cells are embedded in a self-produced extracellular polymeric substance [25,26,27,28]. In this environment, several interactions between species occur, so that the presence of one microorganism is able to create a niche for others, which helps to promote colonization and retention [29,30]. The ability of *Streptococcus mutans* to create very large amounts of glucans, release acid and survive in an acidic environment, combined with its strong binding to teeth, ultimately leads to the disintegration of hydroxyapatite in tooth enamel and dentin [31,32,33,34,35]. Cariogenic bacteria, such as *S. mutans*, can cause degradation of resin composites through bacterial production of acidic derivatives [36,37,38,39], mainly acting at the adhesive tooth-restoration interface, which can limit the functional and esthetic longevity of a composite restoration [40,41,42] resulting in interfacial gaps [43].

Although *S. mutans* has traditionally been seen as the primary cariogenic species, current research appears to suggest that *Candida albicans*, through interactions with *S. mutans*, may also play a significant cariogenic role [44,45,46,47]. Alike *S. mutans*, *C. albicans* is a natural commensal colonizer of the oral cavity [30,48]. However, this opportunistic organism can quickly turn into a pathogen that causes oral candidiasis under conditions of immune suppression or changes in the host environment [49,50,51]. It is well known that *C. albicans* and *S. mutans* interact in the oral cavity in a synergistic way [52,53,54,55,56,57,58] and both physical connections and metabolic interactions have been shown. For example, *S. mutans* uses lactic acid to supply a carbon source for *C. albicans* growth, which in turn causes the oxygen tension to drop to levels favorable to facultative streptococci [59]. *C. albicans* has been described on mineralized tooth surfaces, has shown the ability to adapt in dental biofilms and to possess virulence attributes associated with caries pathogenesis, such as the ability to produce acids, to grow under low pH conditions, combined with a high proteolytic activity [60]. The results of a recent study have clearly shown how the different chemical composition of different light-cured composites subjected to identical surface treatments can determine significant differences in terms of adhesion for *S. mutans* [61]. Similarly, it would be of definite clinical interest to further investigate on the potential of *C. albicans* to adhere and proliferate on the surface of different DRCs, particularly on the surface of composite materials subjected to additional heat-curing protocols aimed at improving their mechanical properties.

Thus, the aim of the present in vitro study was to analyze the ability of *C. albicans* to adhere and form biofilms on the surface of three commercially available heat cured dental composite resins having different chemical formulations but subjected to identical surface treatment and polymerization protocols.

## 2. Materials and Methods

Table 1 shows the experimental groups and composite resins used in the current study design and provides details on the material composition.

### 2.1. Realization of Composite Discs

Disc-shaped specimens were manufactured by placing the gel material in polyvinylsiloxane molds, 2 mm high and having a 4 mm inner hole. To extrude the surplus material, filled molds were positioned between two glass slides held in place with a paper clip. A light-emitting diode curing unit (Celalux 3, VOCO, Cuxhaven, Germany) with an 8 mm tip diameter and an output power of 1300 mW/cm^2^ was used to light-cure the material for 20 s. Each final composite disc had a total surface area of 50.27 mm^2^. An ultrasonic bath was used to clean every disc, which were then subjected to an additional cycle of heat curing in a composite oven at 70 °C for 10 min (Bulb PlusT, Micerium, Avegno, Genova, Italy).

### 2.2. Saliva Collection

Human saliva samples were collected according to D’Ercole et al. (2022) and the Ethics Committee of “G. d’Annunzio” University, Chieti–Pescara, Italy (approval code SALI, N. 19 of the 10 September 2020) [61]. Healthy volunteers were enrolled who had not brushed or flossed their teeth for two hours, without active oral caries, periodontitis, dental care in progress or antibiotic therapy for at least three months before the study start. Saliva was collected, combined, centrifuged (16.000× *g* for 1 h at 4 °C) and filtered through filters with pores of 0.8, 0.45 and 0.2 mm to remove microorganisms. After being incubated for 24–48 h at 37 °C, saliva samples were deemed to be sterile if no growth could be seen in either an aerobic or anaerobic environment [61]. To perform the investigation, sterile saliva was collected into sterile tubes and stored in the freezer. Saliva was used to allow the saliva-acquired pellicle formation on the disks to reproduce a human-like environment.

### 2.3. Microbial Strain

*Candida albicans* S5 clinical strain, isolated from the oral cavity of patients who gave their informed consent for this study (reference number: BONEISTO N. 22-10.07.2021, G. d’Annunzio University, Chieti–Pescara, 10 July 2021), was used for the experiments. *C. albicans* S5, grown on Sabouraud dextrose agar (SAB, Oxoid, Milan, Italy) was cultured in RPMI 1640 (Sigma-Aldrich, Milan, Italy) plus 2% glucose and standardized to Optical Density (OD_600_) = 0.15 corresponding to ≈10^6^ CFU/mL [62]. *C. albicans* S5 was characterized for its capability to form a biofilm on a polystyrene surface, and it was a good biofilm producer.

### 2.4. Experimental Design

All disks, previously sterilized with ultraviolet UV light for 40 min, were placed on 96-well polystyrene microtiter plates and inoculated for 2 h in saliva at 37 °C in a shaking incubator with slight agitation to form the protein pellicle layer on the surface and to provide microbial adhesion. Subsequently, 200 µL of standardized *C. albicans* S5 suspension was added in each well with disk and incubated at 37 °C for 48 h in aerobic atmosphere.

**Figure 1:**
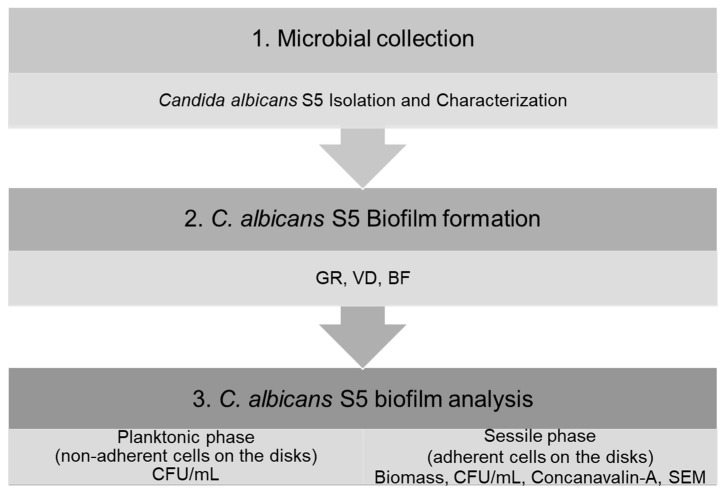
Schematic summary of the study design.

For negative control, each composite disk was inoculated with only medium without the microbial culture and incubated as described above. For each test, a negative control was evaluated. After incubation, the evaluations were performed following the experimental plan (Figure 1).

The planktonic phase was carefully removed from each well, diluted, spread on SAB and incubated at 37 °C for 48 h for the planktonic CFU count (CFU/mL), according to previous studies [55,57]. For the planktonic evaluation, 39 disks (10 tests and 3 negative controls for each different material), in triplicate, for a total of 117 disks were used.

The amount of *C. albicans* S5 adhered to each disk was evaluated for the following:

(i) Sessile CFU count (CFU/mL);
(ii) Biofilm biomass production;
(iii) Extracellular polymeric substances (EPS) of the biofilm matrix;
(iv) Morphology of the sessile colonies by Scanning Electron Microscopy (SEM).

The experimental planning was developed according to D’Ercole et al. [61].

Briefly, for sessile CFU/mL detection, after incubation for 48 h, the adherent viable cells were washed with PBS, detached by ultrasonication and vortexing, spread on SAB plates, incubated for 48 h at 37 °C and counted. The *C. albicans* S5 biomass was determined by Crystal violet (CV) staining at OD_570 nm_. A Concanavalin-A assay was carried out to evaluate the EPS of the biofilm’s matrix. SEM evaluation was performed to morphologically analyze the sessile colonies. After 48 h of in vitro biofilm formation, 5 specimens from each group were fixed for 1 h in 2.5% glutaraldehyde, dehydrated in six ethanol washes (10%, 25%, 50%, 75% and 90% for 20 min and 100% for 1 h) and then dried overnight in a bacteriological incubator at 37 °C. Then, they were coated with gold (Emitech K550, Emitech Ltd., Ashford, Kent, UK) and observed carefully under a SEM (EVO 50 XVP LaB6, Carl Zeiss SMT Ltd., Cambridge, UK) at 15 kV, under 1000× and 5000× magnifications.

For the sessile phase, a total of 294 specimens (10 tests and 3 negative controls for each different material; 117 disks for sessile CFU/mL count and 117 disks for biofilm biomass quantification; 5 disks from each group for Concanavalin A assay and SEM evaluation) were used.

### 2.5. Statistical Analysis

In each group, means and standard deviations were calculated from raw data related to quantitative tests (planktonic CFU count, sessile cells CFU count and biomass quantification by OD_570 nm_). Statistical analysis was carried out according to the analysis of variance (ANOVA) and the Tukey’s test for post hoc intergroup comparisons, using the SPSS for Windows version 21 (IBM SPSS Inc, Chicago, IL, USA). Levene’s and Kolmogorov–Smirnov tests were used to validate the homogeneity of the variances and the normality of data, respectively. The level of α was set at 0.05 for all tests.

## 3. Results

Table 2 summarizes the results achieved from the quantitative tests carried out.

### 3.1. Planktonic CFU/mL Count

**Figure 2:**
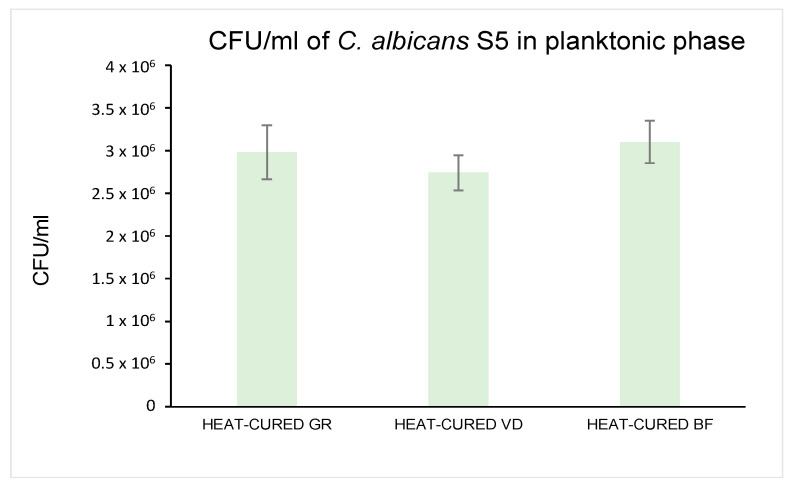
Planktonic *C. albicans* S5 CFU/mL count (×10^5^ CFU/mL).

Figure 2 shows the *C. albicans* S5 CFU/mL count in planktonic phase. Not statistically significant differences were detected among all groups (*p* > 0.05). A slight, but not significant, *C. albicans* S5 CFU/mL reduction was obtained with heat-cured VD (27.40 × 10^5^ ± 2.07 × 10^5^ CFU/mL) as compared to heat-cured GR (29.80 × 10^5^ ± 3.16 × 10^5^ CFU/mL) and BF (31.00 × 10^5^ ± 2.49 × 10^5^ CFU/mL).

### 3.2. Sessile Cells CFU/mL Count

**Figure 3:**
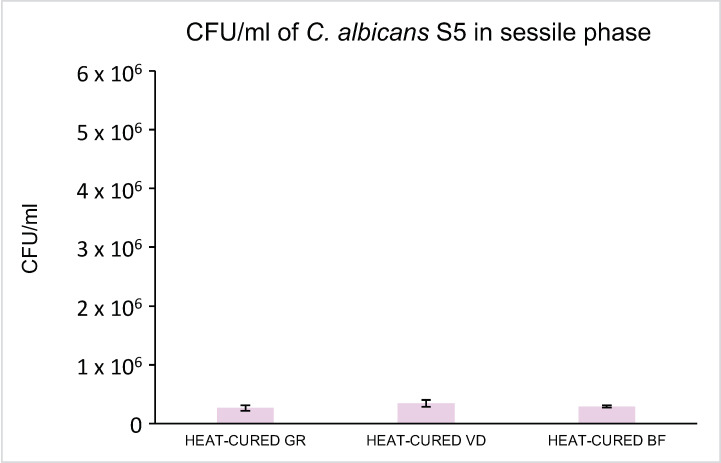
Sessile *C. albicans* S5 CFU/mL count (×10^3^ CFU/mL).

The CFU/mL of *C. albicans* S5 adherent to each tested disk are showed in Figure 3. In detail, the least fungal growth was observed on GR heat-cured (2.65 × 10^5^ ± 0.47 × 10^5^ CFU/mL), with no statistically significant difference (*p* < 0.05) compared to BF heat-cured (2.90 × 10^5^ ± 0.17 × 10^5^ CFU/mL). A significantly increased number of CFU/mL was observed for VD (3.44 × 10^5^ ± 0.56 × 10^5^) (*p* < 0.05).

### 3.3. Biofilm Biomass Quantification by Optical Density (OD_570 nm_)

**Figure 4:**
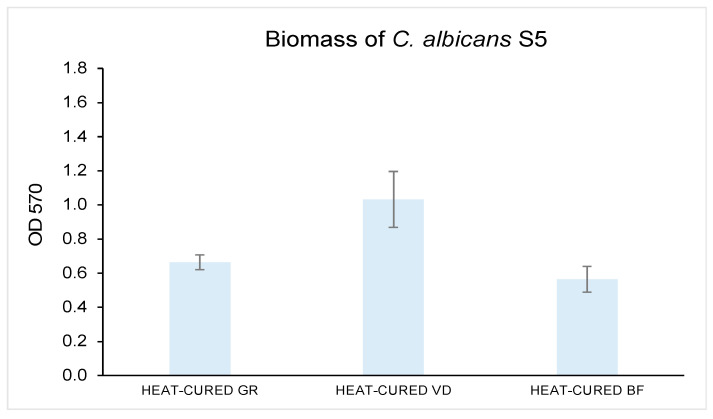
Biomass quantification by optical density (OD_570 nm_).

The biomass production of *C. albicans* S5 adherent to each tested disk (Figure 4) showed a trend similar to that observed for the sessile cells CFU/mL count. The best results were displayed by heat-cured BF, with an OD_570 nm_ of the produced biofilm biomass equal to 0.564 ± 0.0752, significantly reduced as compared to both heat-cured GR (0.6637 ± 0.0427) (*p* < 0.05) and VD (1.0327 ± 0.1642) (*p* < 0.05). The biofilm biomass quantification on GR was significantly lower than VD (*p* < 0.05).

### 3.4. Concanavalin Assay

**Figure 5:**
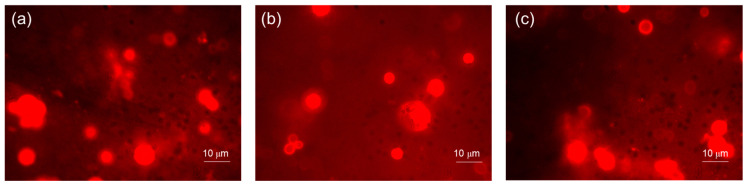
Representative images (original magnification 100×) of polysaccharide matrix (EPS) production by *C. albicans* S5 biofilms observed using Concanavalin-A on heat-cured GR (**a**), heat-cured VD (**b**) and heat-cured BF specimens (**c**).

Figure 5 shows the polysaccharide matrix (EPS) production by *C. albicans* S5 biofilms on different composites disks. The images confirmed the biofilm biomass results with a major production of carbohydrates displayed on a heat-cured VD disk as compared to the other composites. A slight EPS reduction was also observed when comparing heat-cured BF to heat-cured GR.

### 3.5. SEM Analysis

**Figure 6:**
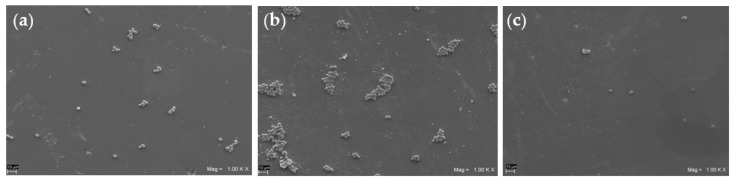
Representative SEM images (original magnification 10,000×) of *C. albicans* S5 biofilm formed on disk-shaped specimens from heat-cured GR (**a**), heat-cured VD (**b**) and heat-cured BF (**c**) groups.

Figure 6 shows representative SEM microphotographs of the *C. albicans* S5 biofilm formation on the composite disks. The VD group, which contained the greatest amount of fungal cells on its surface, showed relatively large adherent aggregates after 48 h of in vitro biofilm development (Figure 6b), while GR and BF showed smaller aggregates and an apparent reduced biofilm formation.

## 4. Discussion

In the present investigation, the behavior of *C. albicans* S5 in the presence of three commercially available, nanohybrid DCRs, coated with human saliva, was evaluated by assessing the planktonic and sessile growth modes, the biofilm biomass quantification and the qualitative analysis of the extracellular polymeric substances (EPS) of the biofilm matrix by the Concanavalin-A assay and SEM observation. All samples were polymerized using the same standardized heat-curing protocol and received the same surface treatment. This made it possible to exclude the impact of possible confounding factors and allowed us to properly assess the effect of the only one variable under investigation (i.e., the unique chemical formulation of each different resin) on the biofilm formation [63,64]. Focusing on planktonic CFU assay, a not statistically significant difference among the tested groups emerged. A slight, but not significant, *C. albicans* S5 planktonic CFU/mL reduction was observed with VD as compared to GR. Regarding the sessile cells CFU count, a significant increase was observed for VD compared to GR and BF. The current findings demonstrated a relevant antiadhesive effect for GR and BF, with a significant decrease in the number of sessile CFUs, in the biomass quantification and in the presence of EPS matrix. BF had the most promising behavior when compared to the other composite, both in terms of antiadhesive activity and due to a general decrease in the biofilm biomass quantification. The *C. albicans* capability to adhere to disk is affected by the nature of the surface, the molecules involved in quorum sensing and the production of adhesin [65]. In particular, Park et al. demonstrated that the *C. albicans* adhesion on resin was reduced by the electrostatic repulsion force created and hydrophobic interactions between the surface and yeast [66]. Probably, even in the present study, a decreased yeast cells growth might be related to an increased surface hydrophilicity. As already reported by Kim et al., cells grown under bis-GMA showed significantly increased surface hydrophobicity, which could potentially enhance the ability of *C. albicans* S5 to adhere to hydrophobic surfaces [67]. As a result, in the current investigation, both bis-GMA-based composites (GR and VD), when compared to a bis-GMA free material (BF), showed a slight rise in the sessile CFU count.

Of course, antiadhesive and antibacterial qualities need to be carefully considered together with any potential cytotoxicity brought on by the monomers present in (and released from) dental materials [68,69]. Numerous in vitro investigations have shown that the free methacrylate monomer residues left over after the polymerization phase, which may cause the production of prostaglandin E2 (PGE2), the expression of cyclooxygenase 2 and an activation of the proinflammatory response via an increase in interleukin-1 (IL-1), IL-6 and nitric oxide (NO), are primarily responsible for the potential cytotoxicity of the CR organic components [70,71]. It has also been observed that resin monomers can affect cellular physiology and adaptive cell responses by boosting ROS generation [72,73]. In a recent study, De Angelis et al. investigated the biological effect of different resin-based composites on human gingival fibroblast by means of flow cytometry analysis and immunofluorescence. Interestingly, based on their results, GrandioSo (Voco GmbH) and Enamel Plus HRi Biofunction (Micerium) seemed the least cytotoxic among the materials they tested, leading to reduced oxidative stress and fewer genotoxic effects [74]. Therefore, for the abovementioned resins (GR and BF), the antiadhesive action and the general reduction in biomass quantification herein observed seems even more promising and clinically relevant, if combined with the reduced cytotoxicity shown in previous studies [70,71,72,73]. Furthermore, as far as GrandioSo (Voco GmbH) is concerned, in a recent study on light-cured materials, D’Ercole et al. found a stronger capability to reduce *S. mutans* growth (in terms of sessile CFU count, biofilm biomass, metabolic activity and polysaccharide production) compared to that of Venus Diamond (Kulzer GmbH), somehow strengthening the clinical relevance of the present findings [61]. In fact, several studies provided striking evidence that *S. mutans* and *C. albicans* develop a symbiotic relationship that enhances the virulence of cospecies plaque biofilms formed on tooth surfaces, ultimately amplifying the severity of disease [53,56,57]. Such an association between *C. albicans* and *S. mutans* appears to be largely mediated by a physical interaction that relies on the production of glucans, which are produced by bacterial exoenzymes (Gtfs), on yeast and hyphal cell surfaces. These interactions are essential for the assembly of an EPS-rich matrix, the formation of enlarged microcolonies containing densely packed *S. mutans* cells and the development of cospecies biofilms [45]. For these reasons, it should be underlined how the ability suggested for GR to limit the proliferation of both *C. albicans* and *S. mutans* might definitely be promising.

A lower amount of biofilm matrix, with a reduced biofilm thickness and less carbohydrate amount accumulation, may be clinically advantageous, as a biofilm with a reduced biomass is supposed to be less resistant to the salivary buffering effect, antibacterial agents and to the action of fluoride ions [61]. The presence of a thick and stable plaque biofilm, on the contrary, makes those defense mechanisms less effective, compromising the long-term outcome of composite resin restorations [43] and potentially increasing the risk for secondary caries. Thus, from a clinical point of view, it would be advisable to select a material that impairs adhesion, proliferation or even just extracellular matrix production of pathogenic microorganisms, in particular (based on the results of the present study) when dealing with indirect composites subjected to post-polymerization thermal cycles.

Among the limitations of the present study, it should be underlined that only the heat-curing protocol was herein evaluated, without taking into account the simple light-curing procedure. Photoactivation is the most prevalent method used to activate and promote the polymerization of resin based dental composites. The irradiance emitted by different models/brands of light-curing devices is variable, usually ranging between 400 and 1200 mW/cm^2^ [75]. However, as already mentioned, monomer conversion is never complete during the polymerization of light-cured resin composites. This is the reasons why, when dealing with indirect restorations, resin composites are conveniently subjected to further heat curing. Such an additional heat treatment leads to an increase in the degree of conversion and improved mechanical properties such as microhardness, flexural strength, fracture toughness, wear resistance, tensile strength, color stability and a reduction of wear [11,12,13,14]. Thus, it seemed of greater clinical interest to investigate the antimicrobial activity of materials when they are used in their best performing modes.

As already mentioned, several studies have reported high prevalence for *S. mutans* in dental biofilms where *C. albicans* resides, suggesting that such a fungal–bacterial interaction can potentially contribute to dental caries [25,26,44,45]. Considering this association between *C. albicans* S5 and *S. mutans*, further studies seem necessary to better investigate the combined susceptibility of both microorganisms to resin-based material formulation in terms of adhesion and proliferation.

In brief, based on the present results, it can be concluded that different formulations of commercially available heat-cured resin composites may interact differently with *C. albicans*. In comparison to VD, GR and BF led to a decreased biofilm growth in terms of sessile CFU count, biofilm biomass and polysaccharide synthesis. Despite the fact that they also permitted *C. albicans* development to a certain level, these characteristics would suggest that GR and BF as more suitable restorative materials. Understanding that heat-cured composites may show a different affinity with *Candida albicans*, depending on their intrinsic chemical composition and despite the surface treatment, may be of great clinical relevance. In fact, an informed clinician could wisely select those materials that are able to limit the formation of biofilms by potentially cariogenic species, which might lead to secondary caries over time. Moreover, a better understanding about the affinity between restorative materials and cariogenic species would also encourage manufacturers of the dental industry towards the production and commercialization of resin composites that are less prone to allow an intense microbial proliferation.

## Figures and Tables

**Table 1:** Composite resins included in this study.

Experimental Group	Material	Manufacturer	Batch	Composition
**GR**	GrandioSO - Shade A2 - (Nanohybrid)	Voco GmbH (Cuxhaven, Germany)	2028459	89% (*w*/*w*) fillers (1 μm glass ceramic filler, 20 nm–40 nm silicon dioxide fillers), Bis-GMA, Bis-EMA, TEGDMA.
**VD**	Venus Diamond - Shade A2 - (Nanohybrid)	Kulzer GmbH (Hanau, Germany)	E8292	71% (*w*/*w*) fillers (0.2 μm Si-Zr fillers), Bis-GMA, TEGDMA.
**BF**	Enamel Plus HRi Biofunction - Shade BF2 - (Nanohybrid)	Micerium (Avegno, Genova, Italy)	2021006247	74% in weight (60% in volume) fillers (0.005 μm–0.05 μm silicon dioxide fillers), (0.2–3.0 μm glass fillers), Urethane dimethacrylate, Tricyclodecane dimethanol dimethacrylate.

**Table 2:** C. albicans S5 detection on the three resin composites investigated.

	Experimental Group		
	GR	VD	BF
Planktonic CFU count (×10^5^ CFU/mL) (SD)	29.80 ^a^	27.40 ^a^	31.00 ^a^
	(3.16)	(2.07)	(2.49)
Sessile Cells CFU count (×10^3^ CFU/mL) (SD)	2.65 ^b^	3.44 ^a^	2.90 ^b^
	(0.47)	(0.56)	(0.17)
Biomass quantification OD_570 nm_ (SD)	0.6637 ^b^	1.0327 ^a^	0.5641 ^c^
	(0.0427)	(0.1642)	(0.0752)

Different superscript letters indicate statistically significant differences.

## Data Availability

The data presented in this study are available on request from the corresponding author.
